# Beyond BMI: the complex interplay of reward sensitivity, eating behaviors, and BMI in female college students

**DOI:** 10.3389/fpsyg.2025.1698965

**Published:** 2025-11-17

**Authors:** Madhawi Aldhwayan, Shahd Alabdulkader, Alexander Dimitri Miras, Shadeena Alhusan, Khloud Alghmdi, Hanan Albarqi, Zainab Almousa, Rasha Alshaalan

**Affiliations:** 1 Department of Community Health Science, College of Applied Medical Sciences, King Saud University, Riyadh, Saudi Arabia; 2 Department of Health Sciences, College of Health and Rehabilitation Sciences, Princess Nourah bint Abdulrahman University, Riyadh, Saudi Arabia; 3 School of Medicine, Ulster University, Derry, United Kingdom

**Keywords:** reward sensitivity, body mass index, eating behavior traits, cognitive restraint, disinhibition, hunger, progressive ratio task

## Abstract

**Background:**

Heightened reward sensitivity (RS) may lead to greater preference for high-calorie foods, excessive intake, and weight gain. However, the association between RS, eating behavior traits, and body mass index (BMI) remains unclear.

**Objective:**

We examined the relationship between RS and BMI and explored the associations of eating behavior traits with RS among female college students.

**Methods:**

A cross-sectional study was conducted with female students aged 18–25 years in Riyadh, Saudi Arabia. Anthropometric measurements were obtained to calculate BMI. Eating behaviors were assessed using the Three-Factor Eating Questionnaire, which measures cognitive restraint, disinhibition, and hunger. RS was evaluated using the progressive ratio task. Correlations between BMI, RS, and eating behavior traits were analyzed using non-parametric statistical methods.

**Results:**

The data of 89 students were analyzed. No significant associations were found between BMI and RS. Similarly, there were no significant correlations between RS and any of the eating behavior traits. Among the Three-Factor Eating Questionnaire subscales, only disinhibition was significantly positively correlated with BMI (*r* = 0.21, *p* < 0.05). RS (breakpoint) was positively correlated with hunger (*r* = 0.25, *p* < 0.05) and negatively correlated with cognitive restraint (*r* = −0.40, *p* < 0.001). There were no significant differences in breakpoint scores between participants with low and high BMI.

**Conclusion:**

Although no significant associations were observed between BMI and RS or between RS and eating behavior traits, the findings contribute to understanding the complex interplay between psychological and behavioral factors in eating and weight regulation.

## 1 Introduction

Reward sensitivity (RS) is a personality trait that controls responsiveness to rewarding stimuli. It is mediated by the behavioral activation system, which drives approach behaviors toward positive reinforcement and avoidance of punishment (Schag et al., 2021; Sutton et al., 2022). The mesolimbic dopamine pathways, including the ventral tegmental area, nucleus accumbens, and orbitofrontal cortex regions, regulate reward anticipation, motivation, and reinforcement learning (Liu and Kanoski, 2018; Loxton, 2018). Along with neurobiological mechanisms, RS is mediated by multiple interacting factors. Psychological traits such as impulsivity, cognitive restraint, and emotional eating have been linked to overeating and weight gain (Kidd and Loxton, 2018; Loxton and Tipman, 2017). Physiological and genetic influences, including dopaminergic receptor polymorphisms and hormonal responses to food cues, also modulate reward-related eating (Portella et al., 2021; Stover et al., 2023). Moreover, social and environmental factors such as cultural traditions (Yanaoka et al., 2022), stress (Hanson et al., 2021), and exposure to obesogenic food environments shape eating patterns and reward-driven food choices (Maxwell et al., 2017).

In the context of eating behavior, individuals with heightened RS often exhibit stronger motivational drives toward rewarding stimuli, such as palatable foods, which can influence eating patterns and weight status (Kidd and Loxton, 2018; Loxton and Tipman, 2017). Neuroimaging studies using functional magnetic resonance imaging (fMRI) further demonstrate that individuals with obesity exhibit enhanced activation in reward-related brain regions when exposed to food cues, reflecting heightened neural reward sensitivity (Alabdulkader et al., 2024; Richter et al., 2023). These findings underscore the role of brain reward circuitry in obesity and highlight the overlap between behavioral and neural mechanisms underlying food motivation.

Eating behavior traits represent key psychological dimensions that influence how individuals regulate food intake in response to internal and external cues. The Three-Factor Eating Questionnaire (TFEQ) is a well-established instrument that captures three major eating behavior constructs: cognitive restraint (conscious restriction of food intake to control body weight), disinhibition (tendency to overeat in response to stress or palatable food cues), and hunger (susceptibility to internal appetite cues) (Stunkard and Messick, 1985). These traits interact with reward processes and have been linked to variations in body mass index (BMI) and dietary control. Specifically, higher disinhibition and hunger scores are often associated with greater BMI, while cognitive restraint may mitigate overeating (Almuhammadi and Alfawaz, 2024; Esposito et al., 2021; Kruger et al., 2016).

Earlier studies assessed RS primarily through self-reported questionnaires; however, recent work has increasingly adopted behavioral measures, such as the progressive ratio task (PRT), a computer-based paradigm used to quantify the motivational drive for rewards, including food (Bell and DeWall, 2019). Bell and DeWall (2019) employed the PRT to investigate how impulsivity and self-regulatory depletion influence reward motivation. In their first study, impulsive individuals expended greater effort to obtain rewards when depleted, though this effect was not replicated in a larger pre-registered study. These findings highlight the PRT’s potential as a behavioral measure of effort-based RS. The PRT has also been applied in studies examining changes in RS following obesity surgeries, where altered responsiveness to food rewards was observed postoperatively, supporting its value in assessing motivational aspects of eating behavior (Abdeen et al., 2019; Althukair et al., 2024).

Understanding how RS interacts with eating behaviors may provide insight into the mechanisms underlying overeating and inform targeted obesity prevention strategies, particularly in at-risk populations such as young adults. Therefore, we aimed to (1) examine the association between RS, as assessed with the PRT, and BMI among female college students and (2) investigate how eating behavior traits influence RS.

## 2 Methods

### 2.1 Study design and participants

This analytical cross-sectional study was conducted in a female-only college in Riyadh, Saudi Arabia between December 2022 and February 2023. A total of 98 female college students aged 18–25 years participated. The cross-sectional design was chosen to explore the relationship between RS, eating behaviors, and BMI. Ethical approval was obtained from the Institutional Review Board of Princess Nora bint Abdulrahman University (HAP-01-R-059).

Participants were recruited via convenience sampling. Posters were distributed across the college to invite participation. Interested students were screened for eligibility and invited to the laboratory for participation. The sample size was calculated *a priori* using G*Power version 3.1.9.4 for correlation analysis. The parameters were set as follows: anticipated effect size (*ρ* H1) = 0.30, *α* error probability = 0.05, power (1 − *β*) = 0.80, and null hypothesis correlation (ρ H0) = 0. The analysis indicated that a minimum of 84 participants was required. Accordingly, 98 participants were recruited to ensure adequate statistical power. Pregnant or lactating women and those who lacked understanding of the task instructions, had known food allergies, had a chronic disease (e.g., thyroid disorders, diabetes mellitus, cardiovascular disease), self-reported diagnosed eating disorders, or were taking medication that may affect appetite (e.g., antibiotics, chemotherapy drugs, codeine, morphine, corticosteroids, cyproheptadine, and tricyclic antidepressants) were excluded. Comprehension of task instructions was assessed by counting the number of candies left and the number of earned candies in the task output.

### 2.2 Data collection

Eligible participants were invited to the nutrition laboratory at the college, and all study procedures were conducted in person. A registered dietitian explained the study protocol and obtained participants’ consent by having them click “I agree” on the iPad questionnaire. Participants were asked to report the time and content of their last meal. Caloric intake was estimated was estimated using the myfood24 online dietary assessment tool (Carter et al., 2015). This information was collected to account for possible internal state effect, which could influence performance on the PRT. All participants completed the PRT at varying post-ingestive periods, without experimental manipulation of fasting status, as the study’s focus was on the relationship between BMI and RS rather than the influence of internal physiological states. Last-meal data were categorized by time since last meal (< 1 h, 1–3 h, > 3 h) and estimated caloric intake (fasting, < 150 kcal, 150–500 kcal, > 500 kcal). Participants then proceeded with the study procedures.

#### 2.2.1 Anthropometric measures

Height and weight were measured using a calibrated digital scale (Seca™, Germany). Measurements were conducted following standard World Health Organization (1995) protocols for anthropometry, with participants measured in light clothing and without shoes to ensure accuracy and reliability. The data obtained were used to calculate BMI by dividing weight in kilograms by height in meters squared. The World Health Organization body mass categorization was used as follows: a BMI less than 19.9 kg/m^2^ indicated underweight, between 20 and 24.9 kg/m^2^ indicated normal weight; between 25 and 29.9 kg/m^2^ indicated overweight; and more than 30 kg/m^2^ indicated obesity.

#### 2.2.2 PRT

The PRT is a computer-based tool that determines the maximum effort a participant will expend by gradually increasing the number of responses required to receive a food reward. Participants were seated in front of a computer screen with a bowl containing 20 candies (M&Ms.® crispy candies, Mars UK Limited, Slough, UK), each providing 4 kcal (43.7% sugars, 44.1% fat). A prompt on the screen instructed them: *“You can earn food by clicking the mouse button. Click as much or as little as you like. When you no longer wish to continue, press the spacebar to stop the session.”* After each ratio was completed, a message appeared stating: *“You have earned food. Enjoy your reward, and after you have swallowed it completely, you may click OK to continue.”* Once the candy was consumed, participants pressed the OK button only if they chose to proceed to the next ratio to earn another candy. The geometric progression schedule applied in this task was selected based on prior experiments (Abdeen et al., 2019; Althukair et al., 2024; Miras et al., 2012). The breakpoints were assessed by the total number of mouse clicks in the last completed ratio, and this served as an index of individuals’ RS.

#### 2.2.3 TFEQ

Participants were asked to complete the TFEQ to assess eating behaviors. The questionnaire consists of 51 items divided into three subscales: cognitive restraint (21 items), disinhibition (16 items), and hunger (14 items). Each item was scored 0 or 1 according to standardized instructions, with higher scores indicating greater expression of the respective trait (e.g., greater dietary restraint, tendency to overeat, susceptibility to hunger cues; Supplementary material 1). The total score for each subscale was obtained by summing the item scores within the subscale. All three subscales were analyzed. The TFEQ has been widely validated and used in diverse populations, including young adults (Stunkard and Messick, 1985).

### 2.3 Statistical analysis

SPSS software version 29.0 (IBM Corp., Armonk, NY, USA) was used for data analysis. Incomplete questionnaires were excluded from the analysis. Categorical variables were expressed as percentages and frequencies, while continuous variables were expressed as mean ± standard deviation or median and interquartile range. The Shapiro–Wilk test was used to evaluate the normality of the data. BMI was stratified into two categories by combining underweight (*n* = 11) and normal weight (*n* = 44) into one group (low BMI: < 25 kg/m^2^, *n* = 55) and overweight (*n* = 25) and obesity (*n* = 9) into another group (high BMI: ≥ 25 kg/m^2^, *n* = 34). This classification was used to ensure sufficient statistical power for comparisons, as the sample sizes for individual BMI categories were relatively small. The Mann–Whitney U test was used to compare the means of the breakpoint, restraint, disinhibition, and hunger between the two groups. Spearman’s correlation test was used to assess the relationship between BMI, breakpoint, and the three TFEQ subscales. Partial Spearman correlations were computed by regressing RS and TFEQ subscales separately on last meal time and caloric intake to control for participants’ internal state. A *p*-value ≤ 0.05 was considered statistically significant.

## 3 Results

The participants’ general characteristics are presented in Table 1. Nine participants were excluded because they did not comprehend the study instructions, leaving a final sample of 89. All participants were women aged 19–25 years, with a mean age of 20.56 ± 1.2 years and a BMI of 23.5 ± 5.1 kg/m^2^. The proportion of participants with a BMI ≥ 25 and < 25 was 38.2% (*n* = 34) and 61.8% (*n* = 55), respectively. Almost half of the participants (46.1%) had their last meal more than 3 h before the study, and 24.7% had their last meal less than 1 h before the study. Regarding calories in the last meal, more than half of the participants (55.1%) consumed 150–500 calories, whereas only 7.9% were fasting.

**Table 1:** Participant characteristics (*N* = 89).

Variable	All	BMI < 25 kg/m^2^	BMI ≥ 25 kg/m^2^
	*N* = 89	*n* = 55 (61.8%)	*n* = 34 (38.2%)
Age (years)	20.56 ± 1.2	20.44 ± 1.1	20.76 ± 1.3
BMI (kg/m^2^)^*^	23.5 ± 5.1	20.3 ± 2.3	28.7 ± 4.0
Time since last meal *n* (%)			
< 1 h	22 (24.7%)	14 (25.5%)	8 (23.5%)
1–3 h	26 (29.2%)	17 (30.9%)	9 (26.5%)
> 3 h	41 (46.1%)	24 (43.6%)	17 (50%)
Calories in last meal *n* (%)			
Fasting	7 (7.9%)	5 (9.1%)	2 (5.9%)
**<** 150 kcal	22 (24.7%)	20 (36.4%)	2 (5.9%)
150–500 kcal	49 (55.1%)	25 (45.5%)	24 (70.6%)
> 500 kcal	11 (12.4%)	5 (9.1%)	6 (17.6%)

BMI, body mass index; kcal: kilocalories. ^*^Significant difference between groups at *p* < 0.05. Values for categorical variables are presented as *n* (%).

Table 2 presents the results of the Mann–Whitney U test comparing the breakpoint from the PRT and the scores of the TFEQ subscales between the high and low BMI groups. Participants with a BMI < 25 kg/m^2^ had a median breakpoint of 80.0 (interquartile range: 40.0–320.0), comparable to those with a BMI ≥ 25 kg/m^2^ [80.0 (interquartile range: 29.0–160.0); *U* = 838.0, *p* = 0.41]. Similarly, no significant differences were observed in restraint, disinhibition, and hunger scores.

**Table 2:** Median (IQR) scores of PRT reward sensitivity and TFEQ eating behavior traits by BMI category.

Variable	BMI < 25 kg/m^2^	BMI ≥ 25 kg/m^2^	Mann–Whitney U test	*p*-value
	*n* = 55	*n* = 34		
Breakpoint	80.0 (40.0–320.0)	80.0 (29.5–160.0)	838.0	0.41
Restraint	8.0 (6.0–11.0)	9.0 (8.0–12.3)	1108.0	0.14
Disinhibition	6.0 (4.0–7.0)	7.0 (4.0–9.0)	1099.0	0.16
Hunger	7.0 (5.0–9.0)	6.5 (4.8–8.0)	919.0	0.120

Values are presented as median (interquartile range). The Mann–Whitney U test was used for group comparisons. BMI, body mass index; TFEQ, Three-Factor Eating Questionnaire; PRT, progressive ratio task.

Table 3 summarizes the correlation matrix of BMI, breakpoint score, and TFEQ subscales. A weak negative relationship (*r* = −0.10, *p* = 0.35) was observed between BMI and breakpoint, which was not statistically significant. A weak positive correlation was observed between BMI and hunger (*r* = 0.25, *p* = 0.02), whereas all other correlations were non-significant. Positive correlations were observed among the TFEQ subscales, indicating an interrelated pattern of eating behavior traits. Correlations between last-meal variables (time since last meal, caloric intake) and RS were non-significant (all *p* > 0.1), suggesting that internal state prior to the PRT did not confound the primary outcomes.

**Table 3:** Spearman’s rank correlation coefficient matrix of BMI, breakpoint, and TFEQ subscale scores.

Variable	BMI	Breakpoint	Restraint	Disinhibition	Hunger
BMI	1	−0.109	0.19	0.21^*^	0.03
Breakpoint	−0.101	1	−0.40^**^	0.04	0.25^*^
Restraint	0.19	−0.40^**^	1	0.19	0
Disinhibition	0.21^*^	0.04	0.187	1	0.36^**^
Hunger	0.03	0.25^*^	0	0.36^**^	1

Values represent Spearman’s correlation coefficient (r) and corresponding *p*-value. BMI, body mass index; TEFQ, Three-Factor Eating Questionnaire. ^*^Correlation is significant at 0.05; ^**^Correlation is significant at 0.01.

## 4 Discussion

We examined the association between BMI and RS, and assessed the association of eating behaviors with RS in a sample of female college students in Saudi Arabia. Our findings suggest that there is no significant association between BMI and RS, as measured by the breakpoint score of the PRT. Similarly, there was no difference in breakpoint scores between participants with a BMI < 25 and those with a BMI ≥ 25.

The findings indicate that BMI is not associated with RS, as participants with a BMI ≥ 25 did not differ from those with a BMI < 25 in their motivation to obtain rewards. Consistent with our results, Jonker et al. (2019) reported that adolescents with obesity and those with a healthy weight did not differ in RS. Similarly, in a prospective cohort study, Jonker et al. (2016) reported no association between RS and BMI among adolescents with overweight and obesity. These studies used performance-based measures indexing attention for cues signaling reward and punishment. Neuroimaging evidence suggests that obesity is associated with heightened brain activity to high-calorie food cues (Alabdulkader et al., 2024; Richter et al., 2023). For example, a functional magnetic resonance imaging study reported that individuals with obesity exhibited greater activation in reward- and flavor-processing brain regions in response to chocolate odors compared with cucumber odors, indicating increased neural sensitivity to energy-dense foods (Han et al., 2021). The lack of consistency across studies may reflect methodological differences, particularly in RS assessment. The use of different tasks, such as behavioral paradigms, neuroimaging, and self-reported measures contribute to the variability in findings. Another possible explanation for our findings is combining participants with overweight and obesity in one group, which may have diluted the potential effect of BMI on RS. Finally, although participants’ last-meal data were collected to account for potential satiety effects on PRT performance, this factor was not experimentally controlled for. Therefore, variation in satiety state may have altered RS results, and this should be considered in interpreting the findings.

Cognitive restraint, defined as the conscious restriction of food intake to control weight, also showed complex associations. Previous research has reported inconsistent findings, with some studies showing increased dietary restraint with lower BMI (Nakamura et al., 2021), while others report increased dietary restraint with higher BMI (Almuhammadi and Alfawaz, 2024; Ramírez-Contreras et al., 2021). In our study, there was no significant difference in cognitive restraint across BMI groups. However, participants with a BMI ≥ 25 had higher, though nonsignificant, restraint scores. Additionally, we observed a significant negative association between cognitive restraint and RS. This finding aligns with prior studies reporting that higher restraint may reduce the rewarding value of food (Watson et al., 2021). Individuals with high cognitive restraint may intentionally avoid rewarding foods to control weight (Adams et al., 2019), but this does not necessarily translate into reduced calorie intake (Burger and Stice, 2011; Chmurzynska et al., 2021). In fact, higher restraint among individuals with obesity has been associated with minimal weight loss and even future weight gain (Burger and Stice, 2011; Nakamura et al., 2021). This paradox may reflect the interplay of restraint with disinhibition, as high restraint combined with high disinhibition often undermines weight control efforts (Watson et al., 2021).

Disinhibited eating reflects a tendency to overeat in response to stress or external food cues. Previous studies have reported higher disinhibition scores with higher BMI (Brunner et al., 2021; Dakin et al., 2023; Kruger et al., 2016), suggesting that individuals with overweight or obesity are more likely to overeat in response to appetitive food cues or stress. For example, Kruger et al. found that each unit increase in disinhibition score was associated with a 0.4 kg/m^2^ increase in BMI and a 0.82% increase in body fat percentage among young adult women (Kruger et al., 2016). Similarly, Legget et al. (2023) reported that higher scores on TFEQ disinhibition and hunger subscales were associated with increased ratings of high-calorie food appeal and desire to eat among women.

In contrast to these findings, we observed non-significant differences in breakpoint and disinhibition scores between participants with low and high BMI. However, higher disinhibition scores were associated with higher BMI. Earlier research also suggests that individuals with higher BMI often display elevated levels of both disinhibition and RS, which together increase the risk of excess energy intake and obesity (Almuhammadi and Alfawaz, 2024; Brunner et al., 2021; Dakin et al., 2023; Ramalho et al., 2023). Individuals with elevated disinhibition may be more prone to overeating independently of their RS levels (Van Malderen et al., 2018). The discrepancy may reflect individual variability or methodological differences, such as sample size and population characteristics. It is also possible that high disinhibition moderates, rather than directly predicts, the relationship between RS and BMI.

Our findings also demonstrated that higher hunger scores were associated with higher RS. Hunger reflects an individual’s sensitivity to internal appetite cues and is closely linked to reward-driven eating behavior. Previous studies have indicated that increased hunger is associated with increased RS, greater behavioral responsiveness to food cues, and stronger motivation for food consumption (Cassidy and Tong, 2017). Consistent with this, research using the TFEQ has reported higher hunger scores among adults with obesity compared with those without obesity (Kruger et al., 2016) and among women compared with men (Legget et al., 2023).

The intercorrelations among the TFEQ subscales observed in this study (i.e., positive associations between restraint, disinhibition, and hunger) highlight the complex interplay of eating behavior traits, suggesting that these psychological dimensions may co-occur and collectively influence individuals’ responsiveness to food cues and body weight regulation. Additionally, the absence of a significant difference between BMI groups and RS and eating behaviors in our study could be attributed to the limited sample size (*n* = 89), which may have reduced statistical power compared with previous studies with larger cohorts.

Altogether, our findings suggest that while BMI is not directly associated with RS, eating behavior traits such as disinhibition, hunger, and cognitive restraint play essential roles in shaping both RS and BMI. These results underscore the importance of a comprehensive approach to weight management that encompasses both psychological and behavioral factors, in addition to traditional dietary interventions. Enhancing self-regulatory skills, practicing mindfulness, managing stress, and promoting healthier eating behaviors may help reduce the risk of overeating and weight gain in young adult populations.

## 5 Conclusion

Our findings indicated that BMI was not significantly associated with RS. However, we observed a positive correlation between disinhibition and BMI, a positive correlation between hunger and RS, and a negative correlation between cognitive restraint and RS. These results contribute to the growing body of literature on the interplay between psychological traits, eating behaviors, and weight-related outcomes.

Although BMI itself was not directly associated with RS, eating behavior traits showed meaningful associations with both BMI and RS, suggesting that these factors may play a critical role in shaping food-related decision-making and long-term weight management. Despite the strength of the findings, the present study has several limitations. First, BMI is a limited proxy for body weight status and body composition; therefore, the findings should be interpreted with caution, and future research may benefit from incorporating more precise measures of adiposity. Second, the cross-sectional design does not allow for causal inference. Finally, participants’ internal state was not controlled for, which may have influenced PRT performance.

Given the complexity of these relationships, a holistic approach that integrates psychological and behavioral dimensions alongside dietary and lifestyle interventions may be particularly beneficial. Future studies with larger and more diverse samples are warranted to further explore these associations and clarify potential moderating effects. Interventions that target self-regulation, mindful eating, portion control, and stress management may support healthier eating patterns and reduce the risk of overweight and obesity among young adults.

## Data Availability

The raw data supporting the conclusions of this article will be made available by the authors, without undue reservation.
