# Supplementary material for: Beyond BMI: the complex interplay of reward sensitivity, eating behaviors, and BMI in female college students
Source: Front Psychol. 2025 Nov 17;16:1698965. doi: 10.3389/fpsyg.2025.1698965 (PMC12667182; doi:10.3389/fpsyg.2025.1698965)
Supplement: Supplementary file 1 [file Data_Sheet_1.docx]

**Supplementary Material 1**

**Three-Factor Eating Questionnaire**

| Part I |  |  |
| --- | --- | --- |
| 1. When I smell a sizzling steak or see a juicy piece of meat, I find it very difficult to keep from eating, even if I have just finished a meal. | T | 2 |
| 1. I usually eat too much at social occasions, like parties and picnics. | T | 2 |
| 1. I am usually so hungry that I eat more than three times a day. | T | 3 |
| 1. When I have eaten my quota of calories, I am usually good about not eating any more. | T | 1 |
| 1. Dieting is so hard from me because I just get too hungry. | T | 3 |
| 1. I deliberately take small helpings as a means of controlling my weight. | T | 1 |
| 1. Sometimes things just taste so good that I keep on eating even when I am no longer hungry. | T | 2 |
| 1. As I am often hungry, I sometimes wish that while I am eating, an expert would tell me that I have had enough or that I can have something more to eat. | T | 3 |
| 1. When I feel anxious, I find myself eating. | T | 2 |
| 1. Life is too short to worry about dieting. | T | 1 |
| 1. As my weight goes up and down, I have gone on reducing diets more than once. | F | 2 |
| 1. I often feel so hungry that I just have to eat something. | T | 3 |
| 1. When I am with someone who is overeating, I usually overeat too. | T | 2 |
| 1. I have a pretty good idea of the number of calories in common food. | T | 1 |
| 1. Sometimes when I start eating, I just can’t seem to stop. | T | 2 |
| 1. It is not difficult for me to leave something on my plate. | F | 2 |
| 1. At certain times of the day, I get hungry because I have gotten used to eating then. | T | 3 |
| 1. While on a diet, if I eat food that is not allowed, I consciously eat less for a period of time to make up for it. | T | 1 |
| 1. Being with someone who is eating often makes me hungry enough to eat also. | T | 3 |
| 1. When I feel blue, I often overeat. | T | 2 |
| 1. I enjoy eating too much to spoil it by counting calories or watching my weight. | F | 1 |
| 1. When I see a real delicacy, I often get so hungry that I have to eat right away. | T | 3 |
| 1. I often stop eating when I am not really full as a conscious means of limiting the amount that I eat. | T | 1 |
| 1. I get so hungry that my stomach often seems like a bottomless pit. | T | 3 |
| 1. My weight has hardly changed at all in the last ten years. | F | 2 |
| 1. I am always hungry so it is hard for me to stop eating before I finish that food on my plate. | T | 3 |
| 1. When I feel lonely, I console myself by eating. | T | 2 |
| 1. I consciously hold back at meals in order not to gain weight. | T | 1 |
| 1. I sometimes get very hungry late in the evening or at night. | T | 3 |
| 1. I eat anything I want, any time I want. | F | 1 |
| 1. Without even thinking about it, I take a long time to eat. | F | 2 |
| 1. I count calories as a means of controlling my weight. | T | 1 |
| 1. I do not eat some foods because they make me fat. | T | 1 |
| 1. I am always hungry enough to eat at any time. | T | 3 |
| 1. I pay a great deal of attention to changes in my figure. | T | 1 |
| 1. While on a diet, if I eat a food that is not allowed, I often then splurge and eat other high-calorie foods. | T | 2 |

| Part II | **1** | | **2** | | **3** | | **4** | |  |  |
| --- | --- | --- | --- | --- | --- | --- | --- | --- | --- | --- |
| 1. How often are you dieting in a conscious effort to control your weight? | 0 | | 0 | | 1 | | 1 | | + | 1 |
| 1. Would a weight fluctuation of 5lbs affect the way you live your life? | 0 | | 0 | | 1 | | 1 | | + | 1 |
| 1. How often do you feel hungry? | 0 | | 0 | | 1 | | 1 | | + | 3 |
| 1. Do your feelings of guilt about overeating help you control your food intake? | 0 | | 0 | | 1 | | 1 | | + | 1 |
| 1. How difficult would it be for you to stop eating halfway through dinner and not eat for the next four hours? | 0 | | 0 | | 1 | | 1 | | + | 3 |
| 1. How conscious are you of what you are eating? | 0 | | 0 | | 1 | | 1 | | + | 1 |
| 1. How frequently do you avoid “stocking up” on tempting foods? | 0 | | 0 | | 1 | | 1 | | + | 1 |
| 1. How likely are you to shop for low-calorie foods? | 0 | | 0 | | 1 | | 1 | | + | 1 |
| 1. Do you eat sensibly in front of others and splurge alone? | 0 | | 0 | | 1 | | 1 | | + | 2 |
| 1. How likely are you to consciously eat slowly in order to cut down on how much you eat? | 0 | | 0 | | 1 | | 1 | | + | 1 |
| 1. How frequently do you skip dessert because you are no longer hungry? | 1 | | 1 | | 0 | | 0 | | - | 3 |
| 1. How likely are you to consciously eat less than you want? | 0 | | 0 | | 1 | | 1 | | + | 1 |
| 1. Do you go on eating binges though you are not hungry? | 0 | | 0 | | 1 | | 1 | | + | 2 |
| 1. On a scale of 0 to 5, where 0 means no restraint (eating whatever you want, whenever you want it) and 5 means total restraint (constantly limiting food intake and never “giving in”), what number would you give yourself? | 0 | 0 | | 0 | 1 | 1 | | 1 | + | 1 |
| 1. To what extent does this statement describe your eating behavior? “I start dieting in the morning, but because of any number of things that happen during the day, by evening I have given up and eat what I want, promising myself to start dieting again tomorrow.” | 0 | | 0 | | 1 | | 1 | | + | 2 |

**SCORING THREE-FACTOR EATING QUESTIONNAIRE**

One point is given for each item in Part 1 and for each item (numbered question) in Part II. The correct answer for the true/false item is indicated in the scoring box and beside it is the number for the factor that it measures. Note: Gray shaded blocks have “false” as the positive answer. The direction of the question in Part II is determined by splitting the responses at the middle. If the item is labeled “+,” those with responses above the middle are given a zero. Vice versa for those with a “-.” For example, anyone scoring 3 or 4 on the first item in Part II (No. 37) would receive one point, anyone scoring 1 or 2 would receive a zero. Note the only reverse scored item is No. 47, shaded in gray

| **1. Restraint** | **2. Disinhibition** | **3. Hunger** |
| --- | --- | --- |
| 4 | 1 | 3 |
| 6 | 2 | 5 |
| 10 | 7 | 8 |
| 14 | 9 | 12 |
| 18 | 11 | 17 |
| 21 | 13 | 19 |
| 23 | 15 | 22 |
| 28 | 16 | 24 |
| 30 | 20 | 26 |
| 32 | 25 | 29 |
| 33 | 27 | 34 |
| 35 | 31 | 39 |
| 37 | 36 | 41 |
| 38 | 45 | 47 |
| 40 | 49 |  |
| 42 | 51 |  |
| 43 |  |  |
| 44 |  |  |
| 46 |  |  |
| 48 |  |  |
| 50 |  |  |
| **1. Total** | **2. Total** | **3. Total** |
